# Lack of *miR-379/miR-544* Cluster Resists High-Fat Diet-Induced Obesity and Prevents Hepatic Triglyceride Accumulation in Mice

**DOI:** 10.3389/fcell.2021.720900

**Published:** 2021-08-30

**Authors:** Congcong Cao, Peng Duan, Wencun Li, Yang Guo, Jin Zhang, Yaoting Gui, Shuiqiao Yuan

**Affiliations:** ^1^Institute of Reproductive Health, Tongji Medical College, Huazhong University of Science and Technology, Wuhan, China; ^2^Guangdong and Shenzhen Key Laboratory of Male Reproductive Medicine and Genetics, Institute of Urology, Peking University Shenzhen Hospital, Shenzhen-Peking University – The Hong Kong University of Science and Technology Medical Center, Shenzhen, China; ^3^Department of Obstetrics and Gynaecology, Xiangyang No. 1 People’s Hospital, Hubei University of Medicine, Xiangyang, China; ^4^The Second Affiliated Hospital, College of Medicine, Zhejiang University, Hangzhou, China; ^5^College of Pharmacy, Hubei University of Medicine, Shiyan, China; ^6^Shenzhen Huazhong University of Science and Technology Research Institute, Shenzhen, China

**Keywords:** miR-379/miR-544, obesity, liver, mouse, knockout, high-fat diet

## Abstract

Non-alcoholic fatty liver disease (NAFLD) affects obesity-associated metabolic syndrome, which exhibits hepatic steatosis, insulin insensitivity and glucose intolerance. Emerging evidence suggests that microRNAs (miRNAs) are essential for the metabolic homeostasis of liver tissues. Many hepatic miRNAs located in the miR-379/miR-544 cluster were significantly increased in leptin-receptor-deficient type 2 mice (db/db), a mouse model of diabetes. However, the function of the miR-379/miR-544 cluster in the process of hepatic steatosis remains unclear. Here, we report that the novel function of miR-379/miR-544 cluster in regulating obesity-mediated metabolic dysfunction. Genetical mutation of miR-379/miR-544 cluster in mice displayed resistance to high-fat diet (HFD)-induced obesity with moderate hepatic steatosis and hypertriglyceridemia. *In vitro* studies revealed that silencing of *miR-379* in human hepatocellular carcinoma (HepG2) cells ameliorated palmitic acid-induced elevation of cellular triglycerides, and overexpression of *miR-379* had the opposite effect. Moreover, *Igf1r* (Insulin-like growth factor 1 receptor) and *Dlk1* (Delta-like homolog 1) were directly targeted by *miR-379* and *miR-329*, respectively, and elevated in the livers of the *miR-379/miR-544* cluster knockout mice fed on HFD. Further transcriptome analyses revealed that the hepatic gene expressions are dysregulated in *miR-379/miR-544* knockout mice fed with HFD. Collectively, our findings identify the *miR-379/miR-544* cluster as integral components of a regulatory circuit that functions under conditions of metabolic stress to control hepatic steatosis. Thus, this miRNA cluster provides potential targets for pharmacologic intervention in obesity and NAFLD.

## Introduction

Non-alcoholic fatty liver disease (NAFLD) is one major cause of chronic liver disease in the world and characterized by excessive triglyceride accumulation in the liver clinically and pathologically (Adams et al., 2005; Samuel et al., 2010; Samuel and Shulman, 2012). The disease is closely related with many kinds of metabolic disorders, such as obesity, type 2 diabetes (T2D), and dyslipidemia (Younossi et al., 2011; Ajmal et al., 2014). NAFLD in rodents is generally induced by a high-fat diet (HFD), which causes excess accumulation of triglyceride in the liver and insulin resistance (Wang et al., 2018; Zhang et al., 2018; Zhou et al., 2018). In addition, the liver is a metabolic organ that regulates metabolism of glucose and lipid, and many hepatic genes have been found essential for the development of fatty liver and insulin resistance (Postic et al., 2004). Although the pathogenesis of NAFLD has been studied extensively, the mechanism underlying is still not fully uncovered yet.

MicroRNAs (miRNAs) are a class of endogenous small non-coding RNAs (ncRNA), which could silence gene expression at the post-transcriptional level through imperfect complementary binding with the 3′-untranslated regions (UTRs) of the target mRNAs (Ambros, 2004; Bartel, 2004). Importantly, *miR-24, miR-30c, miR-33, miR-122*, and *miR-130* have been found as key regulators of lipid metabolism (Wen and Friedman, 2012; Ramirez et al., 2013; Soh et al., 2013; Ng et al., 2014; Xiao et al., 2015). Previous microarray screening showed that the *miR-379, miR-411, miR-299*, and *miR-543* were upregulated in the liver of hyperglucocorticoidemia and obesity (db/db mouse model) as well as human liver in a GC/GR-dependent manner (de Guia et al., 2015). These upregulated miRNAs are all located in the *miR-379/miR-544* genomic cluster with high conservation in mammalian species (Glazov et al., 2008), which resides on the human and mouse chromosomes 14 and 12, respectively. Another group reported that the level of serum *miR-379* was significantly up-regulated in NAFLD patients compared to normal controls (Okamoto et al., 2020). Moreover, hepatocyte-specific silencing of *miR-379* remarkably reduced the level of circulating very-low-density lipoprotein (VLDL)-associated triglyceride (TG) in healthy mice and normalized unusual lipid profiles in metabolically impaired animals (de Guia et al., 2015). Previous studies have suggested that deficiency of the *miR-379/miR-544* cluster led to CLPG-like muscular hypertrophy (Gao et al., 2015), but no studies have linked this miRNA cluster to hepatic lipid accumulation and metabolic dysfunction.

The Delta-like homolog 1 (DLK1) is a single transmembrane protein homologous to the Notch pathway ligand Delta but lacks typical Notch interaction sequences (Schmidt et al., 2000; Takada et al., 2000), and has been reported to regulate nutrient metabolism and protect from steatosis (Charalambous et al., 2014). DLK1 is expressed in multiple embryonic tissues before birth, but only in preadipocytes after birth (Schmidt et al., 2000). Ectopic overexpression of DLK1 resulted in a lipodystrophic phenotype related to triglyceride accumulation and glucose intolerance (Lee et al., 2003; Villena et al., 2008). In addition, insulin-like growth factor 1 (IGF1) / insulin-like growth factor 1 receptor (IGF1R) signaling pathway has been demonstrated to be essential for the process of insulin resistance, which leading to metabolic diseases and type 2 diabetes (Fernandez et al., 2001; Le Roith et al., 2002). Mice with one of the *Igf1r* alleles deficient (*Igf1r*^±^) globally exhibited a 10% decrease in post-natal growth, and developed glucose intolerance and insulin resistance with age (Bokov et al., 2011). However, the underlying molecular links among DLK1, IGF1/IGF1R signaling pathway, and *miRNA-379/miR-544* cluster in obesity-mediated metabolic dysfunction, like NAFLD, are mostly poorly understood.

Here, we identified *miR-379/miR-544* cluster as the critical regulator to resist HFD-induced obesity and regulate moderate hepatic steatosis via targeting *Igf1r* and *Dlk1* directly. Although knockout *miR-379/miR-544* cluster in mice do not exhibit overt abnormalities except for muscular hypertrophy in this study, we found that *miR-379/miR-544* knockout mice (KO) offer resistance to HFD-induced obesity and moderate hepatic steatosis. Furthermore, we demonstrated that *miR-329* and *miR-379*, which belong to the *miR-379/miR-544* cluster, could directly target *Igf1r* and *Dlk1*, respectively, and repress their expression. *In vitro* studies also showed that silencing of *miR-379* in HepG2 cells ameliorated palmitic acid (PA)-induced elevation of cellular triglycerides. Further transcriptome analyses revealed that the hepatic gene expressions are dysregulated in *miR-379/miR-544* KO mice fed with HFD by RNA-seq. Our data identify a novel function of the *miR-379/miR-544* cluster in regulating liver steatosis and metabolic syndrome and provide a clue of this miRNA cluster as a potential therapeutic target for obesity and NAFLD.

## Results

### Upregulation of *miR-379/miR-544* Cluster in the Steatotic Livers of db/db Mice and HFD-Fed Mice

**FIGURE 1:**
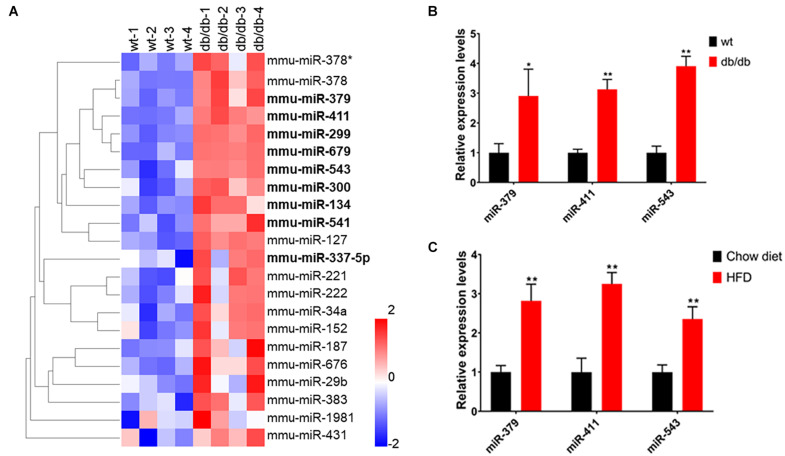
The *miR-379/miR-544* cluster expression is upregulated in the steatotic liver of db/db mice and HFD-fed mice. **(A)** Heatmap showing relative miRNA expression between wild-type (wt) and db/db mice (*n* = 4). The higher and lower expression is showed with red and blue color, respectively. Nine significantly upregulated miRNAs (*miR-379, miR-411, miR-299, miR-679, miR-543, miR-300, miR-134, miR-541*, and *miR-337-5p*) located in the *miR-379/miR-544* cluster are shown with bold font. Differentially regulated miRNAs are defined with fold changes ≥ 2-fold, *P* < 0.05. **(B,C)** Hepatic expression levels of *miR-379, miR-411*, and *miR-543* were determined by real-time PCR in chow-fed 10-12-week-old db/db mice **(B)** and WT mice (*n* = 4) fed an HFD for 10 weeks starting at 4 weeks of age **(C)**. *P < 0.05 and **P < 0.01.

A previous study has shown that many hepatic miRNAs were up- or down-regulated more than two-fold in db/db diabetic mice compared to wild-type (WT) mice by microarray (de Guia et al., 2015). To explore whether the *miR-379/miR-544* cluster is involved in regulating hepatic metabolism, we performed miRNA expression profiling of livers from WT and db/db mice using their microarray data. The results showed that the expression levels of nine miRNAs (*miR-379, miR-411, miR-299, miR-679, miR-543, miR-300, miR-134, miR-541*, and *miR-337-5p*) located in the *miR-379/miR-544* genomic cluster were significantly upregulated in db/db mice compared to WT mice (Figure 1A and Supplementary Table 1). Then we verified the expression of three miRNAs (*miR-379, miR-411*, and *miR-543*) selected from 9 upregulated miRNAs by RT-qPCR in the steatotic livers of db/db mice and mice fed a high-fat diet, respectively. Compared with the respective control mice, db/db mice and mice fed with HFD displayed significantly increased expression levels of hepatic *miR-379, miR-411*, and *miR-543*, consistent with microarray results (Figures 1B, C). In addition, RT-qPCR revealed that most of the miRNAs (such as *miR-299, miR-300, miR-495, miR-544*, etc.) in the *miR-379/miR-544* cluster were significantly elevated in the livers of db/db mice and mice fed with HFD compared with controls (Supplementary Figures 1A,B). Interestingly, except for in livers, we also found that the expression levels of the primary transcript of the *miR-379/miR-544* cluster in white adipose tissue (WBT) of HFD-feeding mice were significantly increased compared to that of control mice (Supplementary Figure 2A). These data suggest that *miR-379/miR-544* cluster might be involved in regulating hepatic metabolism in mice.

### Spatiotemporal Expression Pattern and Genetic Ablation of the *miR-379/miR-544* Cluster in Mice

**FIGURE 2:**
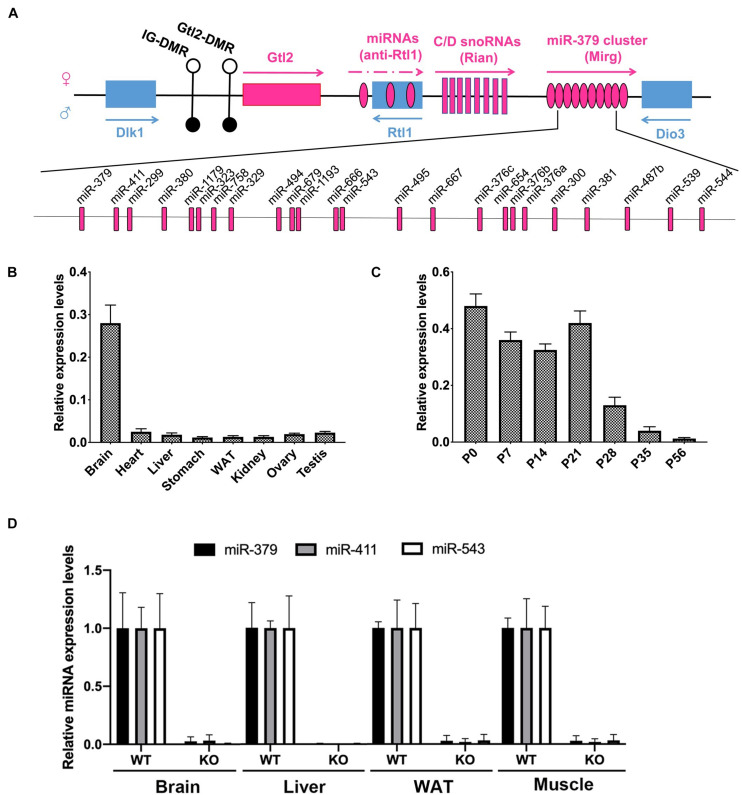
Expression of the *miR-379/miR-544* cluster in multiple tissues and developing livers in mice. **(A)** Schematic representation of *miR-379/miR-544* cluster at the imprinted *Dlk1-Dio3* region on the mouse distal chromosome 12 (human 14q32). **(B)** Expression pattern of the *pri-miR-379/miR-544* cluster transcript in eight different adult mouse tissues (*n* = 4), assayed via quantitative PCR. **(C)** Expression pattern of the *pri-miR-379/miR-544* cluster transcript in developing mouse livers (*n* = 4). Livers at postnatal day 0 (P0, newborn), P7, P14, P21, P28, P35, and P56 were analyzed. **(D)** Expression of selected miRNAs scattered with miRNA cluster (*miR-379, miR-411*, and *miR-543*) in brain, liver, WAT (white adipose tissue), and muscle from WT and the *miR-379/miR-544* cluster KO mice (*n* = 3) at P20 was determined by quantitative PCR.

Since the *miR-379/miR-544* locus is the largest known placental mammal-specific miRNA cluster containing 24 miRNA genes and located within the highly conserved imprinted Dlk1-Dio3 region (Figure 2A), we measured the expression pattern of the *miR-379/miR-544* precursor transcript containing all 24 miRNAs in eight adult different mouse organs using semi-quantitative RT-PCR and quantitative RT-PCR (RT-qPCR). Interestingly, we found that the expression of the primary transcript of the *miR-379/miR-544* cluster was mostly restricted to the adult brain, not in the adult liver (Figure 2B and Supplementary Figure 2B). We then measured the primary transcript levels of the *miR-379/miR-544* cluster in the postnatal developing livers. A higher expression level of the *miR-379/miR-544* cluster transcripts in perinatal livers was observed, whereas lower expression was detected in adult livers (Figure 2C and Supplementary Figure 2C). This expression pattern in the liver was similar to that reported for the *miR-379/miR-544* cluster in testis (Cao et al., 2018). Given that, we assume that the *miR-379/miR-544* cluster plays a role in mouse liver development. To investigate the physiological role of the *miR-379/miR-544* cluster *in vivo*, we generated a knockout (KO) mouse line as our previous report described (Cao et al., 2018). Our breeding strategy was validated by showing the *miR-379, miR-411*, and *miR-543* were not detected by RT-qPCR analysis in several tissues of WT and KO mice at postnatal day 20 (P20) (Figure 2D). The efficiency of *miR-379/miR-544* cluster deletion in liver was further confirmed by semi-quantitative RT-PCR (Supplementary Figures 2D,E). The analyses showed that the primary transcript of the *miR-379/miR-544* cluster had no expression in KO mice liver at P20, suggesting that we successfully deleted *miR-379/miR-544* KO cluster in mouse liver.

### Genetic Deletion of the *miR-379/miR-544* Cluster Confers Resistance to HFD-Induced Obesity

**FIGURE 3:**
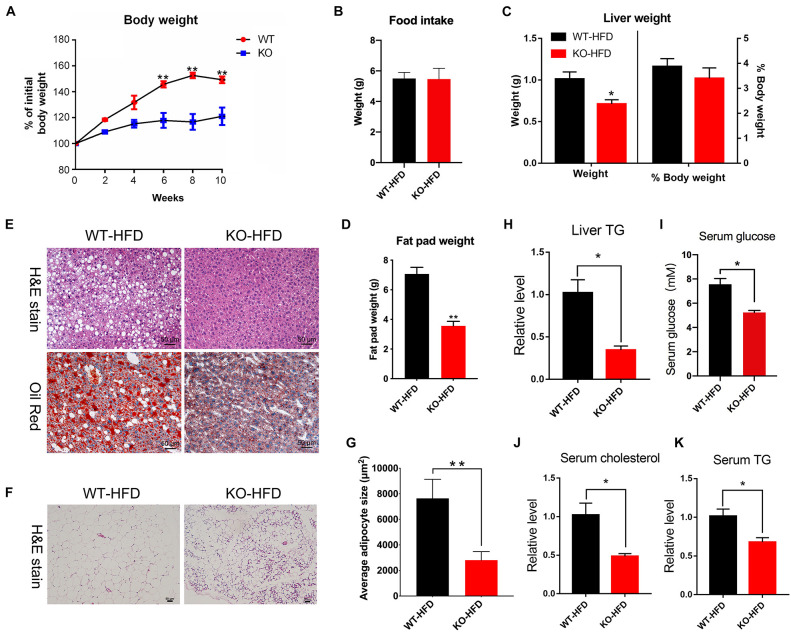
The *miR-379/miR-544* cluster KO mice displayed resistance to HFD-induced obesity. **(A)** The liver growth curve shows that the *miR-379/miR-544* cluster KO-HFD mice gain less weight than WT-HFD mice (*n* = 5). *P < 0.05 and **P < 0.01. **(B)** Measurement of food intake of *miR-379/miR-544* cluster KO-HFD mice and WT-HFD mice (*n* = 3). **(C)** Liver weight and the ratio of liver weight to body weight in the *miR-379/miR-544* cluster KO-HFD mice and WT-HFD mice (*n* = 3). *P < 0.05. **(D)** Representative images of H&E staining (top) and Oil-red O staining (bottom) of liver sections from WT-HFD (left) or the *miR-379/miR-544* cluster KO-HFD (right) mice (*n* = 3). Scale bars = 50 μm. **(E)** Weight of total fat pads isolated from the *miR-379/miR-544* cluster KO-HFD and WT-HFD mice (*n* = 3). **P < 0.01. **(F)** Representative images of H&E-staining of epididymal fat pads from WT-HFD and the *miR-379/miR-544* cluster KO-HFD mice (*n* = 3). Scale bars = 50 μm. **(G)** Quantitation of adipocyte size between WT-HFD and KO-HFD mice as in panel **(F)** are shown (*n* = 3). **P < 0.01. **(H–K)** Measurement of liver TG **(H)**, serum TG **(I)**, random serum glucose **(J)**, and serum cholesterol **(K)** levels in WT-HFD and KO-HFD mice (*n* = 3) are shown, respectively. *P < 0.05.

Since the *miR-379/miR-544* KO mice did not exhibit any overt abnormalities under basal conditions (Cao et al., 2018), we subjected WT and KO mice to metabolic stress by feeding an HFD for 10 weeks to further determine the role of *miR-379/miR-544* in the steatotic liver (hereafter named WT-HFD and KO-HFD mice, respectively). Surprisingly, as shown in Figure 3A, WT mice displayed a significant increase in the body weight after HFD induced for 6 weeks, but KO mice gained much less body weight and were resistant to obesity induced by the HFD. Furthermore, unlike WT-HFD mice, the KO-HFD mice did not develop hepatic steatosis. And food intake remained unaltered in KO-HFD mice, compared with WT-HFD mice (Figure 3B). Interestingly, the weights of KO-HFD mouse livers exhibited a significant reduction compared to WT-HFD, but the ratio of liver weight to the body weight did not change significantly (Figure 3C). Moreover, compared with the WT-HFD mice, the KO-HFD mice had a smaller fat pad weight (Figure 3D). In addition, H&E and Oil Red O staining of hepatic histological sections provided further evidence of reduced liver fat deposition in KO-HFD mice compared with the WT-HFD mice (Figure 3E). H&E staining showed that the epididymal adipose cell size in KO-HFD mice was markedly smaller than that of in WT-HFD mice (Figures 3F,G). Consistent with these histological results, transmission electron microscope (TEM) revealed fewer lipid droplets accumulated in KO-HFD mouse liver than WT-HFD mouse liver (Supplementary Figure 3A). Notably, the levels of liver triglyceride (TG), serum TG, serum glucose, and serum cholesterol in KO-HFD mice were significantly lower than in WT-HFD mice (Figures 3H–K), suggesting global amelioration of the metabolic syndrome that accompanies obesity upon depletion of *miR-379/miR-544* cluster. Together, these data indicate that genetic deletion of the *miR-379/miR-544* cluster could confer resistance to HFD-induced obesity and moderate hepatic steatosis.

### The *miR-379/miR-544* Cluster Deletion Improves HFD-Induced Glucose Tolerance and Whole-Body Insulin Sensitivity

**FIGURE 4:**
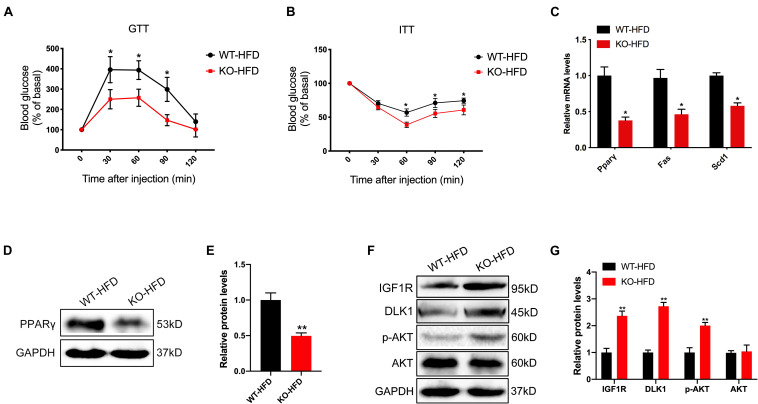
The *miR-379/miR-544* cluster KO mice exhibit reduced obesity-associated insulin resistance after feeding with 10 weeks of HFD. **(A,B)** Blood glucose levels in WT-HFD and KO-HFD mice (*n* = 4) during a glucose-tolerance test (GTT) **(A)** and insulin-tolerance test (ITT) **(B)**. *P < 0.05. **(C)** RT-qPCR analyses of lipogenic genes (*Pparγ, Fas*, and *Scd1*) expression in livers from WT-HFD and KO-HFD mice (*n* = 3). *P < 0.05. **(D,E)** The protein level of PPARγ in livers from WT-HFD and KO-HFD mice (*n* = 3) was determined **(D)** and quantified **(E)** by Western blot analysis. **P < 0.01. **(F,G)** Western blot analysis **(F)** and quantitation **(G)** of IGF1R, DLK1, phosphorylated AKT (p-AKT), and total AKT in the livers from WT-HFD and KO-HFD mice (*n* = 3). **P < 0.01.

In humans and rodents, obesity is frequently associated with alterations in glucose homeostasis and insulin sensitivity. We thus carried out glucose- and insulin-tolerance tests (GTT and ITT) to determine whether glucose tolerance and clearance would be affected by genetic deletion of the *miR-379/miR-544* cluster in mice under the HFD condition. The results showed that the KO-HFD mice exhibited improved glucose tolerance and clearance (Figures 4A,B). In addition, we observed KO-HFD mice exhibited reduced mRNA levels of lipogenesis-related genes (*Ppar*γ, *Fas*, and *Scd1*) via RT-qPCR (Figure 4C). We also measured the protein level of PPARγ in WT-HFD and KO-HFD mouse livers, and the result was consistent with its mRNA level (Figures 4D,E).

Given that *miR-379* could suppress the IGF1/IGF1R and PI3K/AKT signaling pathway in hepatocellular carcinoma (Chen et al., 2016; Huang et al., 2016), and DLK1 is the potential target gene of *miR-329* that may be involved in IGF1 signaling and muscle growth through Akt phosphorylation (Glass, 2005; Nueda et al., 2008). We next measured the protein level of IGFIR, DLK1, and AKT phosphorylation in WT-HFD and the KO-HFD mouse livers. As expected, the expression levels of IGF1R, DLK1, and AKT phosphorylation (Ser 473) in KO-HFD mouse livers were significantly higher than those in WT-HFD mouse livers (Figures 4F,G). Together, these findings strongly indicate that the activation of IGF1/IGF1R and PI3K/AKT signaling pathway contributed to the altered glucose and lipid homeostasis in the *miR-379/miR-544* cluster KO mice fed on HFD.

### Suppression of *miR-379* Expression Ameliorates Palmitic Acid-Induced Lipid Accumulation in HepG2 Cells

**FIGURE 5:**
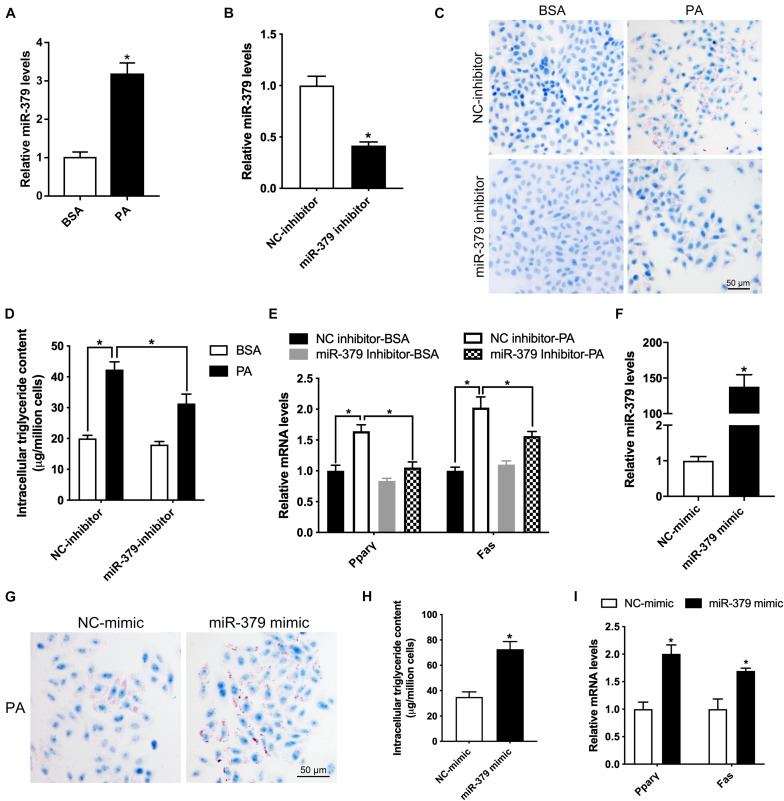
Suppression of *miR-379* expression ameliorates palmitic acid-induced lipid accumulation in HepG2 cells. **(A)** Histogram showing the relative expression of *miR-379* in HepG2 cells (*n* = 4) treated with BSA or 300 μM palmitic acid (PA) for 24 h. *P < 0.05. **(B)** Histogram showing the relative expression of *miR-379* in HepG2 cells (*n* = 3) transfected with *miR-379* inhibitor or negative control (NC) for 48 h. *P < 0.05. **(C–E)** Oil-red O staining **(C)**, intracellular triglyceride content **(D)**, and RT-qPCR analysis of *Ppar*γ and *Fas* **(E)** in HepG2 cells (*n* = 3) transfected with a *miR-379* inhibitor or NC for 48 h in the presence of PA or not. *P < 0.05. Scale bars = 50 μm. **(F)** HepG2 cells (*n* = 3) were transfected with *miR-379* mimic (overexpression) or related negative control (NC) for 48 h, and RT-qPCR analyzed the expression level of *miR-379*. **(G–I)** Oil-red O staining **(G)**, intracellular triglyceride content **(H)**, and RT-qPCR analysis of *Ppar*γ and *Fas* **(I)** in HepG2 cells (*n* = 3) transfected with a *miR-379* mimic or NC for 48 h in the presence of palmitic acid. *P < 0.05.

To further determine the underlying molecular mechanism of the *miR-379/miR-544* cluster in regulating liver steatosis, we utilized the HepG2 cell line as a model to confirm the *in vivo* findings. First, we treated HepG2 cells with 300 μM palmitic acid (PA), a toxic lipid, for 24 h and found that the expression level of *miR-379* increased three-folds (Figure 5A). Then, we added *miR-379* inhibitor for 48 h and found that the expression of *miR-379* was inhibited in HepG2 cells (Figure 5B), accompanied by reduced lipid accumulation and cellular triglyceride content in the presence of PA (Figures 5C,D). Moreover, the suppression of *miR-379* reversed the PA-induced elevation of mRNA expression of Pparγ and Fas (Figure 5E), consistent with the *in vivo* results from *miR-379/miR-544* KO-HFD mouse livers. To further ask whether *miR-379* could induce triglyceride deposition *in vitro*, *miR-379* was overexpressed in HepG2 cell line by transfection of a *miR-379* mimic (Figure 5F). As revealed by Oil Red O staining, the *miR-379* mimic dramatically facilitated lipid accumulation in HepG2 cells (Figure 5G). Similarly, the cellular triglyceride content increased in *miR-379* overexpressed HepG2 cells (Figure 5H). This increase in cellular triglycerides was associated with increased expression of *Ppar*γ and *Fas* (Figure 5I). Together, these *in vitro* data confirmed the reliability of the results obtained from the *miR-379/miR-544* KO-HFD mouse model.

### *Igf1r* and *Dlk1* Are Among the Metabolic Targets of the *miR-379/miR-544* Cluster

**FIGURE 6:**
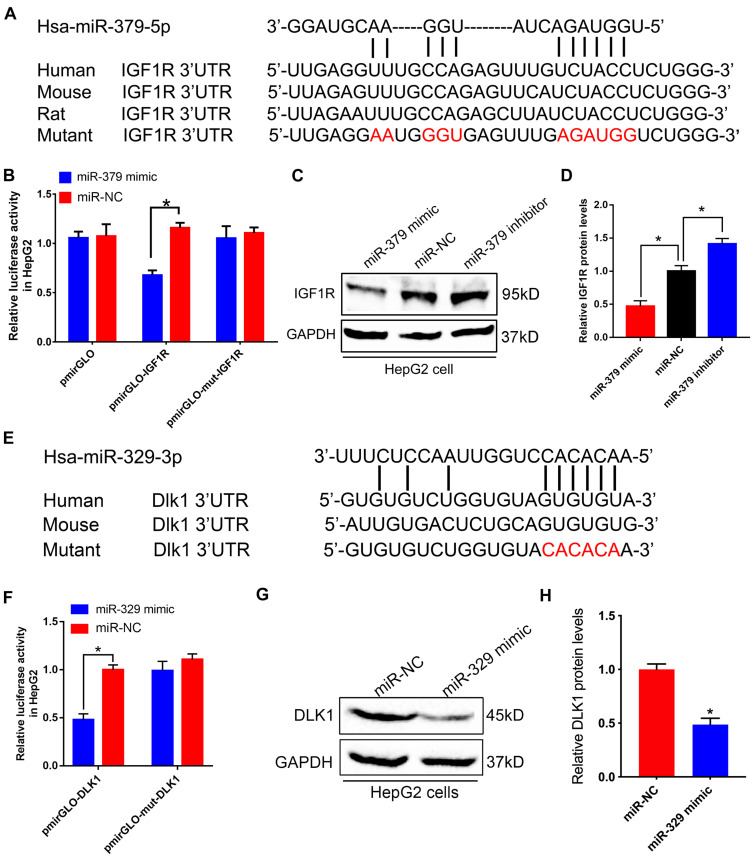
*miR-379/miR-544* cluster directly targets *Igf1r* and *Dlk1*. **(A)** Sequence alignment of human *miR-379-5p* with 3′-UTRs of human, mouse, rat, and mutant *Igf1r*. **(B)** Luciferase reporter assay showing wild-type or mutant *Igf1r* 3′-UTR transfected with *miR-379* mimic or negative control in HepG2 cells (*n* = 3). HepG2 cells transfected with the empty luciferase reporter vector served as control. *P < 0.05. **(C,D)** Western blot **(C)** and quantitative analysis **(D)** of the IGF1R protein compared with GAPDH when HepG2 cells (*n* = 3) were transfected with a miR-379 mimic or a *miR-379* inhibitor. *P < 0.05. **(E)** Schematic representation of potential *miR-329-3p* binding sites and the designed mutant sites at *Dlk1* 3′-UTR is shown. **(F)** Luciferase reporter assay showing wild-type or mutant 3′-UTR transfected with *miR-329* mimic or negative control in HepG2 cells (*n* = 3). *P < 0.05. **(G,H)** Western blot **(G)** and quantitative analysis **(H)** of the DLK1 protein when HepG2 cells (*n* = 3) were transfected with a *miR-329* mimic. *P < 0.05.

Potential effects of *miR-379/miR-544* cluster on glucose and lipid homeostasis promoted us to explore the downstream effectors of these miRNAs. By using the bioinformatics prediction (Lewis et al., 2005), we identified *Igf1r*, a key component of the critical node in the IGF1/IGF1R signaling pathway, contains a potential miRNA response element (MRE) for *miR-379-5p* in its 3′-untranslated region (3′-UTR). This MRE site is highly conserved in humans, mice, and rats (Figure 6A). To investigate whether *Igf1r* could be regulated by *miR-379-5p*, we generated luciferase reporter constructs encoding *Igf1r* wild-type and mutant 3′-UTR sequence, and co-transfected with *miR-379-5p* mimic or negative control into HepG2 cells. Through luciferase reporter assay, we found that luciferase activity with *Igf1r* 3′-UTR was significantly inhibited by *miR-379-5p* mimic, whereas its activity with the *Igf1r* mutated 3′-UTR did not alter significantly (Figure 6B). Moreover, an increase in IGF1R expression occurred in HepG2 cells incubated with a *miR-379* inhibitor (Figures 6C,D), suggesting *miR-379* could inhibit the protein level of endogenous IGF1R in HepG2 cells. Likewise, we found that another miRNA, *miR-329-3p*, located in the *miR-379/miR-544* cluster, has a putative binding site in the 3′-UTR of *Dlk1* (Figure 6E). In addition, like miR-379, the expression levels of hepatic miR-329 in db/db mice and mice fed with HFD were significantly increased compared with that of control mice (Supplementary Figures 3B,C). Luciferase reporter assay revealed that *miR-329* could directly bind to *Dlk1* 3′-UTR, but not to mutant *Dlk1* 3′-UTR (Figure 6F) and repress the protein level of endogenous DLK1 in HepG2 cells (Figures 6G,H). Taken together, these data indicate that *Igf1r* and *Dlk1* are direct target genes of the *miR-379/miR-544* cluster, contributing to regulating the glucose and lipid homeostasis in *miR-379/miR-544* KO-HFD mouse models.

### Genetic Ablation of the *miR-379/miR-544* Cluster in HFD Mice Causes the Dysregulation of Hepatic Gene Expression

**FIGURE 7:**
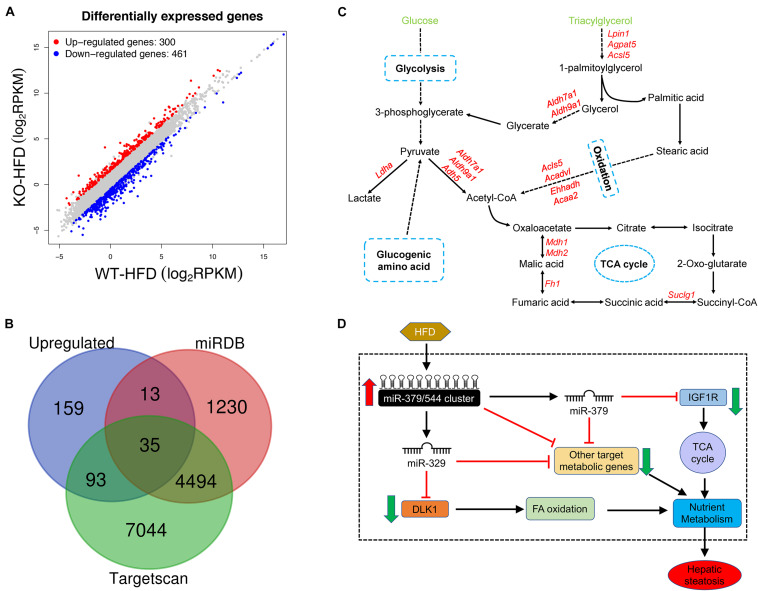
*miR-379/miR-544* cluster KO-HFD mice display dysregulation of hepatic gene expression. **(A)** Volcano plot showing differential expression of 761 significantly altered genes that determined by RNA-seq (*n* = 3). **(B)** Venn diagram showing a comparison between significantly upregulated genes and genes predicted to be *miR-379/miR-544* cluster targets by Targetscan and microCosm. **(C)** Schematic representation of the predicted metabolic genes targeted by *miR-379/miR-544* cluster on glucose and triacylglycerol metabolism processes. **(D)** Schematic diagram of the working model of *miR-379/miR-544* cluster in liver metabolic regulation. *Igf1r*, a transmembrane tyrosine kinase receptor, is a target of *miR-379* that regulates the TCA cycle. *Dlk1* is a target gene of *miR-329* and involves in fatty acid oxidative metabolism. The miR-379/miR-544 cluster is proposed to be an integral component of a regulatory circuit that functions under conditions of metabolic stress to control the overall energy homeostasis.

To gain a better understanding of the molecular mechanisms underlying the specific function of *miR-379/miR-544* cluster in hepatic steatosis, we next performed detailed gene expression analyses in livers of WT-HFD and KO-HFD mice by RNA-seq. The results revealed that a total of 761 genes are significantly deregulated in KO-HFD mouse livers compared to that of WT-HFD mouse livers (Figure 7A and Supplementary Table 2). Among them, 300 genes were upregulated, whereas 461 were genes downregulated in the KO-HFD mice compared to those in WT-HFD mice (Figure 7A and Supplementary Figure 4A). Further RT-qPCR analyses confirmed that 10 randomly selected mRNA expression levels were consistent with the RNA-seq data (Supplementary Figure 4B). We then adopted an alternative approach by analyzing the *miR-379/miR-544* cluster targets predicted by TargetScan (Lewis et al., 2005) and microCosm (Griffiths-Jones et al., 2006) among upregulated genes in RNA-Seq to identify whether the effects on deregulated genes are caused by the primary targets of *miR-379/miR-544* cluster. We found very few overlapping genes, which could indicate that either only a few upregulated genes are the real primary targets or that many targets are relevant only in the specific metabolic pathway (Figure 7B). Furthermore, we performed Sylamer analyses to identify the miRNAs responsible for the dysregulated mRNAs (van Dongen et al., 2008). In general, the enriched seed sequences (“words with the highest peak”) were not that of the *miR-379/miR-544* cluster (Supplementary Figure 4C). These data implied that the transcriptomic changes observed in the *miR-379/miR-544* KO-HFD mice were most likely representing secondary effects.

To further examine whether these differentially expressed genes (DEGs) were related to glucose and lipid homeostasis, we tried to perform Gene Ontology (GO) and Kyoto Encyclopedia of Genes and Genomes (KEGG) analyses using our RNA-seq data. Many upregulated DEGs were enriched for genes involved in metabolic processes through the GO biological process analysis, including oxidation-reduction process, lipid metabolic process, etc. (Figure 3C and Supplementary Table 3). Moreover, the GO biological process analysis of downregulated genes was related to cell differentiation and MAPK cascade (Supplementary Figure 4E and Supplementary Table 4). Through the KEGG pathway analysis, these DEGs were correlated with many metabolic pathways such as non-alcoholic fatty liver disease, glycolysis/gluconeogenesis, and citrate cycle (Supplementary Figures 4F,G and Supplementary Tables 5, 6). In addition, we further analyzed miRNAs target genes that were upregulated in the RNA-Seq data and found many targets that participated in the metabolic processes such as glycolysis, oxidation, and tricarboxylic acid cycle (Figure 7C).

Taken together, our unbiased transcriptome-wide studies uncover a miRNA-controlled gene expression program whose sustained perturbation is very likely to underlie the inhibitors in the setting of obesity observed in the *miR-379/miR-544* cluster KO mice fed on HFD (Figure 7D).

## Discussion

In the current study, we identified a potential function of the *miR-379/miR-544* cluster in regulating nutrient metabolism. First, we found that the expression of many miRNAs located in the *miR-379/miR-544* cluster are significantly upregulated in the liver of mice fed on HFD as well as db/db mice. Second, our *in vivo* results suggested that the *miR-379/miR-544* cluster knockout mice are protected from HFD-induced obesity and alleviated hepatic steatosis. Third, our in vitro data showed that suppression of *miR-379* expression ameliorates palmitic acid-induced lipid accumulation in HepG2 cells, and overexpression of *miR-379* promotes lipid accumulation in HepG2 cells. Finally, among the members of this cluster, we identify *miR-379* and *miR-329* could inhibit the expression of the IGF1R and DLK1 directly via binding with the 3′-UTR of their mRNAs. In addition, the differentially expressed genes between WT and the *miR-379/miR-544* cluster KO mice fed on HFD are involved in metabolic processes, including oxidation-reduction process, lipid metabolic process, and cell differentiation. Interestingly, although the *miR-379/miR-544* mutants did not exhibit other overt phenotypes except CLPG-like muscular hypertrophy under normal laboratory conditions (Gao et al., 2015; Cao et al., 2018), the *miR-379/miR-544* KO-HFD mice displayed resistance to obesity and moderate hepatic steatosis in this study. These consequences broadened the vision of the role of *miR-379/miR-544* under HFD conditions, which supports the notion that the function of miRNAs could be enhanced under conditions of stress (Leung and Sharp, 2010).

Accumulated evidence has showed that the abnormal expression of miRNAs in the liver was associated with the pathogenesis of metabolic disease, including fatty liver disease, type 2 diabetes, and hepatocellular carcinoma (Cheung et al., 2008; Li et al., 2009). Among them, mammalian conserved *miR-379/miR-410* genomic cluster induced in hepatic tissue has been identified as a critical component of GC/GR-driven metabolic dysfunction. Notably, *miR-379* was up-regulated in obesity mouse models in a GC/GR-dependent manner, consistent with our results. Specific silencing of *miR-379* in hepatocytes substantially decreased the levels of triglyceride in healthy mice (de Guia et al., 2015). Using a mouse knockout model, one group identified a deletion of the *miR-379/miR-410* cluster caused abnormalities in energy homeostasis maintenance, and finally partially penetrant neonatal lethality (Labialle et al., 2014). Based on the previous findings, the current study had proposed for the first time that genetic deletion of *miR-379/miR-544* cluster attenuated high fat diet-induced hepatic steatosis and metabolic dysfunction.

Insulin-like growth factor 1 receptor is a tyrosine kinase receptor involved in liver injury and hepatic cell growth (Caro et al., 1988; Scharf et al., 1998) and could regulate (hepatocyte) metabolism indirectly through affecting the development and function of vital metabolic tissues. Moreover, in the liver, IGF1/IGF1R can regulate hormonal inputs via changing extra-hepatocyte function and/or change nutrient flux through altering tissue-specific metabolism of carbohydrate and lipid. These actions are not mutually exclusive, but are used to integrate the functions of all tissues to meet the metabolic needs of the organism (Boucher et al., 2016; Softic et al., 2016; Kineman et al., 2018). Our study showed that upregulation of the IGF1/IGF1R signaling pathway protected the *miR-379/miR-544* cluster KO mice against high-fat diet-induced obesity. Luciferase reporter activity assays confirmed that *Igf1r* was a direct target of *miR-379* in hepatocytes by using *Igf1r* 3-′UTR reporter constructs carrying binding site mutation, in the presence or absence of *miR-379*. Interestingly, a previous study showed that exogenous uptaken of DLK1 attenuated hepatic steatosis, hyperglycemia and glucose intolerance in the diabetic mice (Charalambous et al., 2014). It was also found that the metabolic effects of DLK1, including augmented fatty acid oxidation and decreased gluconeogenesis in the liver, work via AMPK activation (Lee et al., 2016). In our study, we identified *Dlk1* as a direct target of *miR-329*, another member of *miR-379/miR-544* cluster, in hepatocytes in this study. Importantly, similar to the phenotypes of *miR-379/miR-544* KO-HFD mice, both the genetic and the positive energy balance dietary model suggested that the reduction of hepatic lipid synthesis and increase in skeletal muscle lipid oxidation enabled the DLK1-overexpressing mice to be protected from steatosis (Charalambous et al., 2014). Moreover, the production of pituitary growth hormone was elevated in DLK1-overexpressing mice due to a local defect in IGF1 feedback (Charalambous et al., 2014). Interestingly, in our study, we found that when the expression DLK1 was increased, the activation of AKT signaling also increased consistently in the *miR-379/miR-544* cluster KO-HFD mouse livers. Therefore, these results clearly suggested that the *miR-379/miR-544* cluster, which lay within the *Dlk1-Dio3* imprinting region, might regulate the expression of *Dlk1* negatively.

Importantly, increased expression level of *miR-379* was not only found in obesity mouse models of NAFLD and HepG2 cells treated with PA, but also detected in human patients, implying that the level of hepatic *miR-379* correlated with both serum cortisol and TG levels in NAFLD patients and exhibits high potential as a biomarker for NAFLD (de Guia et al., 2015; Okamoto et al., 2020). Moreover, our results showed that the binding sites of *miR-379* within the *Igf1r* 3′-UTRs and *miR-329* within the *Dlk1* 3′-UTRs were conserved between humans and mice, which further indicated the potential of *miR-379/miR-544* cluster as a therapeutic target for human NAFLD. Furthermore, the *miR-379/miR-544* cluster deficiency probably leads to variability in some metabolic genes’ expression and, beyond a certain threshold, to misexpression of some genes by epigenetic regulation, affecting both glucose and lipid metabolism.

In summary, our study illustrated the important role of the *miR-379/miR-544* cluster in the regulation of glucose and lipid homeostasis. Disruption of the *miR-379/miR-544* cluster dosage might have important consequences for obesity and metabolic disease. Our results revealed a previously unknown function of the *miR-379/miR-544* cluster in resistance to high-fat induced obesity and moderate hepatic steatosis, suggesting that the *miR-379/miR-544* cluster might be a potential target for the treatment of lipid-related disorders.

## Materials and Methods

### Mice

The *miR-379/miR-544* cluster knockout (KO) mice generated as described previously (Cao et al., 2018) and maintained under a 12-h dark/light cycle in a specific pathogen-free animal facility. Mice were fed a chow diet with 10% kcal fat (Research Diet D12450B) or a high-fat diet with 60% kcal fat-containing diet (Research Diet D12492) for 10 weeks.

### Glucose and Insulin Tolerance Tests

Glucose-tolerance tests were carried out on mice that had been fasted overnight for 16 h. After determining fasted blood glucose levels, glucose (2 g/kg of body weight) was intraperitoneally injected into each animal. Blood glucose levels were detected after 30, 60, 90, and 120 min. Insulin-tolerance tests were performed on mice following a 5-hour fast. After determining fasted blood glucose levels, animals were injected intraperitoneally with 0.75 U/kg body weight of insulin. Blood glucose levels were detected after 30, 60, 90, and 120 min.

### Measurement of Metabolic Profile

The levels of triglyceride and cholesterol in livers, serum, and cultured cells were determined according to the manufacturer’s instructions (Njjcbio, China). Briefly, 50-100 mg of liver tissue was homogenized in 450-900 ml absolute ethyl alcohol, and centrifuged supernatants were harvested. The extracted contents from the liver, serum, and cells were enzymatically measured with respective kits and were expressed relative to liver weight or serum volume.

### Histological Analysis

Liver tissues, epididymal fat pads, and cultured cells were harvested, fixed in methanol-free 4% paraformaldehyde for paraffin embedding. Tissue specimens were sectioned at 5 μm and then were processed for hematoxylin and eosin (H&E) staining. For oil-red O staining, the subset of fixed livers was sequential 10 and 20% sucrose equilibration for 12 h each, and then cryo-embedded in optimal cutting temperature medium following by 20 μm cryosection cutting with oil red O for evaluating the lipid accumulation. Each image of cultured cells, liver, and fat pad sections was obtained using a light microscope.

### RNA Isolation and Quantitative PCR Analysis

Total RNA was extracted from mouse tissues or cultured cells using Trizol reagent (Invitrogen), according to the manufacturer’s protocol. The purified RNA samples were then quantified with a fluorimeter and reverse transcribed using the PrimeScript RT Reagent Kit with gDNA Eraser (Takara). Then cDNAs were quantified using ABI Prism Step-One System (Applied Biosystems) with SYBR Green Master Mix reagents (Takara). For miRNA detection, total RNA was reverse-transcribed using a miRNA-specific stem-loop primer and subsequently measured by real-time PCR using the miRNA-specific primer. Relative gene expression levels were calculated using the ΔΔCT method (with U6, miR-16, or Gapdh used as the reference gene) and normalized as indicated. The information of primers for quantitative RT-PCR (qRT-PCR) are all listed in Supplementary Table 7.

### Western Blotting Analysis

Protein was extracted from frozen liver tissues and cultured HepG2 cell lines by using RIPA buffer. 30 μg of protein was loaded onto SDS-polyacrylamide gel electrophoresis (PAGE) gel and transferred to PVDF membranes (Bio-Rad). Then the membranes were blocked in 5% non-fat milk (blocking solution) for 1h and incubated with anti-PPARγ polyclonal antibody (1:1000, A0270, ABclonal), anti-AKT antibody (1:1000, no.9272, Cell Signaling Technology), anti–phospho-Akt (Ser 473) antibody (1:1000, no.9271, Cell Signaling Technology), anti-IGF1R polyclonal antibody (1:1000, A0243, ABclonal), anti-DLK1 polyclonal antibody (1:1000, A6578, ABclonal), and anti-GAPDH polyclonal antibody (1:6000, 10494-1-AP, proteinteach) overnight at 4°C. Membranes were washed with TBST three times and incubated with HRP-conjugated goat anti-rabbit IgG (1:5000, 1706515, Bio-Rad) for 1 h, and the immunoreactive bands were visualized with ECL plus Western Detection System (Bio-Rad). Band optical densities were quantified using Image J software (NIH).

### Transmission Electron Microscopy (TEM)

Liver tissues were fixed in 2.5% glutaraldehyde in 0.1 M sodium cacodylate buffer (pH 7.4) for 1 h. After being washed three times with 0.1 M phosphate buffer, the tissues were fixed with 1% osmium tetroxide in 0.1 M phosphate buffer for another 1 h. The samples were dehydrated with increasing ethanol concentration, embedded in epoxy resin, and cut to a thickness of 70 nm. Electron photomicrographs were taken of the ultrastructure of the liver tissues with a TEM (Hitachi, Japan), with the imaging parameters of the lens mode set as zoom-1 HC1, an acceleration voltage of 80.0 kV, a spot size of micro 8 and ×24,500 magnification.

### Cell Culture, Cell Transfections, and Palmitic Acid (PA) Treatment

The HepG2 cell line was cultured in low-glucose Dulbecco’s modified Eagle’s medium (L-DMEM) (HyClone) supplemented with 10% horse serum (HyClone), 100 units/ml penicillin (HyClone), and 0.1 mg/ml streptomycin (HyClone) at 37°C with humidified air and 5% CO_2_. miRNA mimics and inhibitors or the negative control (NC) were purchased from GenePharma (Shanghai). Transfection of miRNAs was performed with HiPerFect transfection reagent (Qiagen). 0.25 M palmitic acid (Sigma) were dissolved in 100% ethyl alcohol. Before using, 0.25 M palmitic acid stock and 5% BSA were incubated in a 60°C water bath for 5-10 min. Then, 320 μl palmitic acid stock was dropwise added into 20 ml of 5% BSA to make 4 mM palmitic acid. Before experiments, 4 mM palmitic acid was incubated in a 60°C water bath for 5-10 min, and a mixture of palmitic acid was added at a total concentration of 300 μM.

### Luciferase Reporter Assay

The annealed oligonucleotides with the WT *Igf1r*-3′UTR binding site or the oligonucleotides with mutated miR-379-5p-binding sites were cloned into pmirGLO control luciferase reporter vector (Promega, Madison, WI, United States). And the annealed oligonucleotides with the WT *Dlk1*-3′UTR binding site or the oligonucleotides with mutated miR-329-3p-binding sites were cloned into pmirGLO control luciferase reporter vector. After being seeded into 96-well plates, HepG2 cells were co-transfected with pmirGLO-*Igf1r* or pmirGLO-mut-*Igf1r* plasmids and *miR-379* mimic or negative control using lipofectamine 2000 (Invitrogen). Meanwhile, pmirGLO-*Dlk1* or pmirGLO-mut-*Dlk1* plasmids and *miR-329* mimic or negative control were also co-transfected into HepG2 cells. Luciferase activity was detected after 2 days of post-transfection using a dual-luciferase reporter kit (Catalog no. E1910; Promega) in which Firefly luciferase activity was normalized against Renilla luciferase activity according to the manufacturer’s instructions.

### RNA-Seq Analysis

Total RNA was extracted from liver tissues of WT-HFD and KO-HFD mice using TRIzol (Invitrogen). The quality of RNA was determined using an Agilent 2100 Bioanalyzer (Agilent RNA 6000 Nano Chip). High-quality total RNA [RNA integrity number (RIN).7] was used as the starting material. All samples were in biological triplicates. RNA-Seq was performed using an Illumina HiSeq ×10 sequencing system according to the manufacturer’s instructions. The DESeq software used negative binomial distribution and a shrinkage estimator for the distribution’s variance to detect differential expression of mRNAs from high-throughput sequencing assay.

### Statistical Analysis

All data are expressed as means ± SEMs. Significant differences were assessed either by a two-tailed Student *t*-test or one-way ANOVA followed by the Student-Newman-Keuls (SNK) test. *P* < 0.05 was considered statistically significant.

## Data Availability Statement

All RNA sequencing data were deposited in the NCBI SRA (Sequence Read Achieve) database with the accession number of PRJNA699696.

## Ethics Statement

The animal study was reviewed and approved by the Institutional Animal Care and Use Committee (IACUC) of the Tongji Medical College, Huazhong University of Science and Technology.

## Author Contributions

SY and CC conceived and designed the research. CC, PD, WL, YGuo, JZ, and YGui performed all bench experiments and data analyses. CC and PD wrote the manuscript. SY revised the manuscript and supervised the project. All authors read and approved the manuscript.

## Conflict of Interest

The authors declare that the research was conducted in the absence of any commercial or financial relationships that could be construed as a potential conflict of interest.

## Publisher’s Note

All claims expressed in this article are solely those of the authors and do not necessarily represent those of their affiliated organizations, or those of the publisher, the editors and the reviewers. Any product that may be evaluated in this article, or claim that may be made by its manufacturer, is not guaranteed or endorsed by the publisher.

## Supplementary Material

Supplementary Figure 1 The expression levels of miRNAs located in the *miR-379/miR-544* cluster were determined by RT-qPCR in the steatotic liver of db/db mice **(A)** and HFD-fed mice **(B)**. *^∗^P* < 0.05 and *^∗∗^P* < 0.01. *n* = 4.

Supplementary Figure 2 Expression of *pri–miR-379/miR-544* cluster transcript in multiple tissues and developing mouse livers. **(A)** The expression level of the *pri–miR-379/miR-544* cluster transcript in multiple tissues from chow-feeding and HFD-feeding mice (*n* = 4). ^∗^*P* < 0.05. **(B)** Expression pattern of the *pri–miR-379/miR-544* cluster transcript in eight different adult mouse tissues (*n* = 4), assayed via semi-quantitative PCR. **(C)** Expression pattern of the *pri–miR-379/miR-544* cluster transcript in mouse livers (*n* = 4) at various postnatal days, assayed via semi-quantitative PCR. **(D,E)** Semi-quantitative RT-PCR **(C)** and quantitative RT-PCR **(D)** analyses of the *pri–miR-379/miR-544* cluster in livers from WT and KO mice (*n* = 3) at postnatal day 20 (P20). *Gapdh* was detected as a control in panels **(A–C)**.

Supplementary Figure 3 **(A)** Representative transmission electron microscopy images of WT-HFD and KO-HFD mouse livers are shown. The red arrows indicate lipid droplets. **(B,C)** The relative expression levels of hepatic *miR-329* in db/db mice and mice fed with HFD are shown, respectively.

Supplementary Figure 4 RNA-seq revealed differential gene expression in KO-HFD mouse livers compared with that of in WT-HFD. **(A)** Heatmap and hierarchical clustering of 761 significantly altered genes in livers from WT-HFD and KO-HFD mice (*n* = 3). The blue-to-red colors of the heatmap are linearly mapped to the Z-scores, which range from −2 to 2. **(B)** The expression levels of 10 selected genes from RNA-seq in WT-HFD and KO-HFD mice livers by RT-qPCR analyses are shown. **(C)** A sylamer plot of motif enrichment in the 3′-UTRs of the dysregulated mRNAs assayed by RNA-seq. **(D)** Gene Ontology (GO) biological process analyses of significantly upregulated differential expressed genes (DEGs). **(E)** GO biological process analyses of significantly downregulated DEGs. **(F)** Kyoto Encyclopedia of Genes and Genomes (KEGG) pathway analyses of significantly upregulated DEGs. **(G)** KEGG pathway analyses of significantly downregulated DEGs.
