# Supplementary figures and images for: Lack of miR-379/miR-544 Cluster Resists High-Fat Diet-Induced Obesity and Prevents Hepatic Triglyceride Accumulation in Mice
Source: Front Cell Dev Biol. 2021 Aug 30;9:720900. doi: 10.3389/fcell.2021.720900 (PMC8435714; doi:10.3389/fcell.2021.720900)

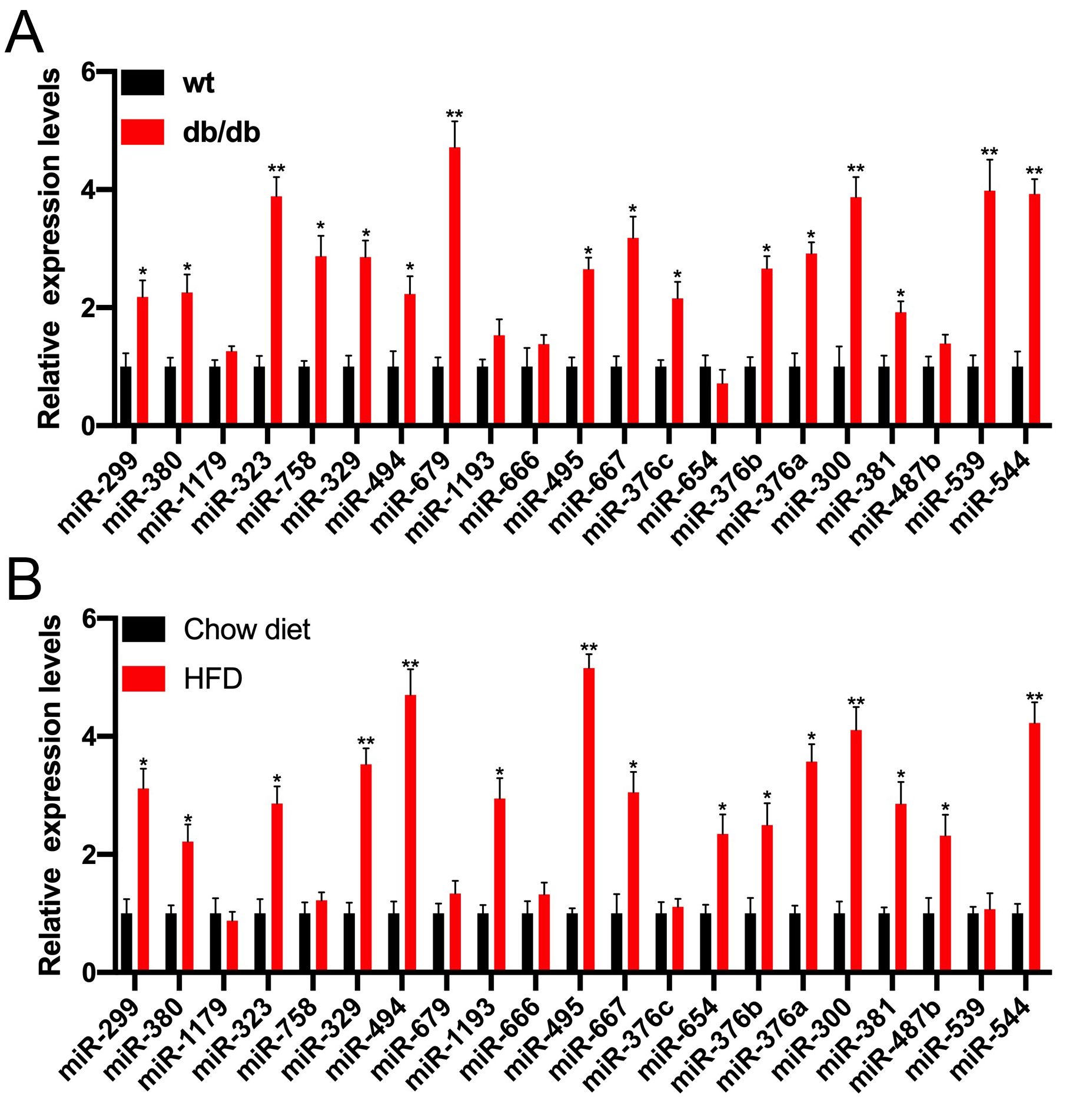

Supplement: Supplementary Figure 1 — The expression levels of miRNAs located in the miR-379/miR-544 cluster were determined by RT-qPCR in the steatotic liver of db/db mice (A) and HFD-fed mice (B). ∗P < 0.05 and ∗∗P < 0.01. n = 4. [file Image_1.TIF]

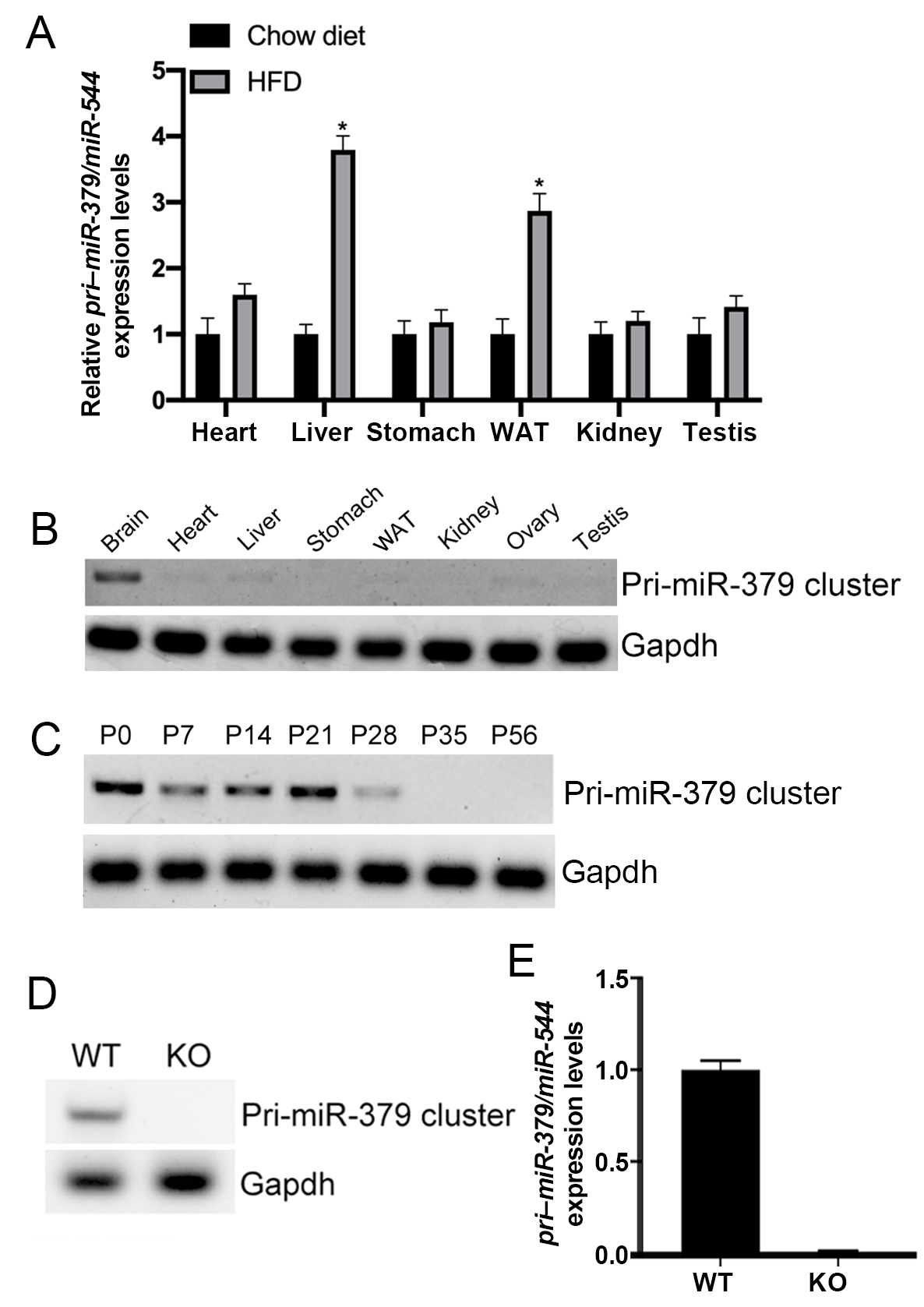

Supplement: Supplementary Figure 2 — Expression of pri–miR-379/miR-544 cluster transcript in multiple tissues and developing mouse livers. (A) The expression level of the pri–miR-379/miR-544 cluster transcript in multiple tissues from chow-feeding and HFD-feeding mice (n = 4). ∗P < 0.05. (B) Expression pattern of the pri–miR-379/miR-544 cluster transcript in eight different adult mouse tissues (n = 4), assayed via semi-quantitative PCR. (C) Expression pattern of the pri–miR-379/miR-544 cluster transcript in mouse livers (n = 4) at various postnatal days, assayed via semi-quantitative PCR. (D,E) Semi-quantitative RT-PCR (C) and quantitative RT-PCR (D) analyses of the pri–miR-379/miR-544 cluster in livers from WT and KO mice (n = 3) at postnatal day 20 (P20). Gapdh was detected as a control in panels (A–C). [file Image_2.TIF]

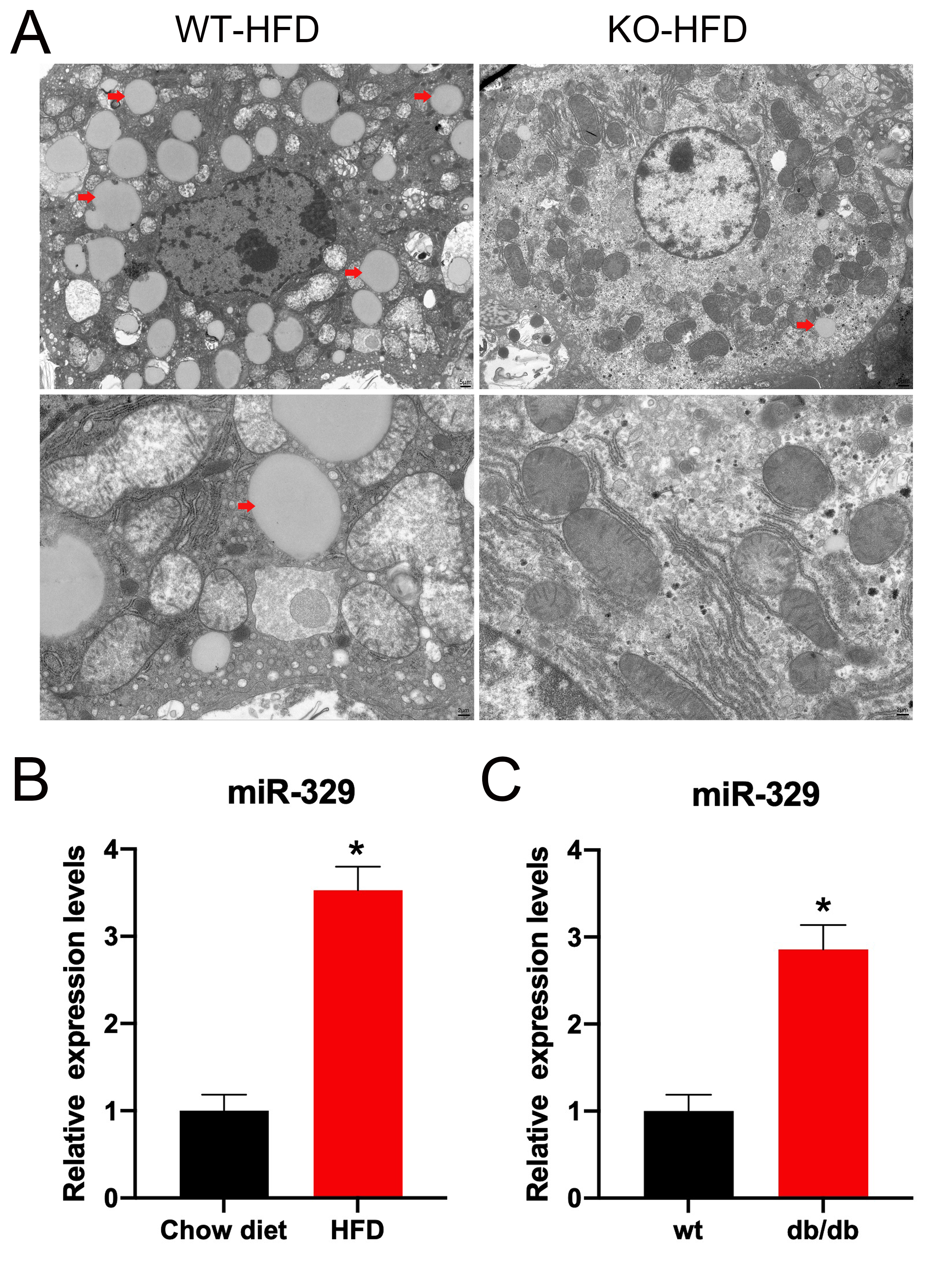

Supplement: Supplementary Figure 3 — (A) Representative transmission electron microscopy images of WT-HFD and KO-HFD mouse livers are shown. The red arrows indicate lipid droplets. (B,C) The relative expression levels of hepatic miR-329 in db/db mice and mice fed with HFD are shown, respectively. [file Image_3.TIF]

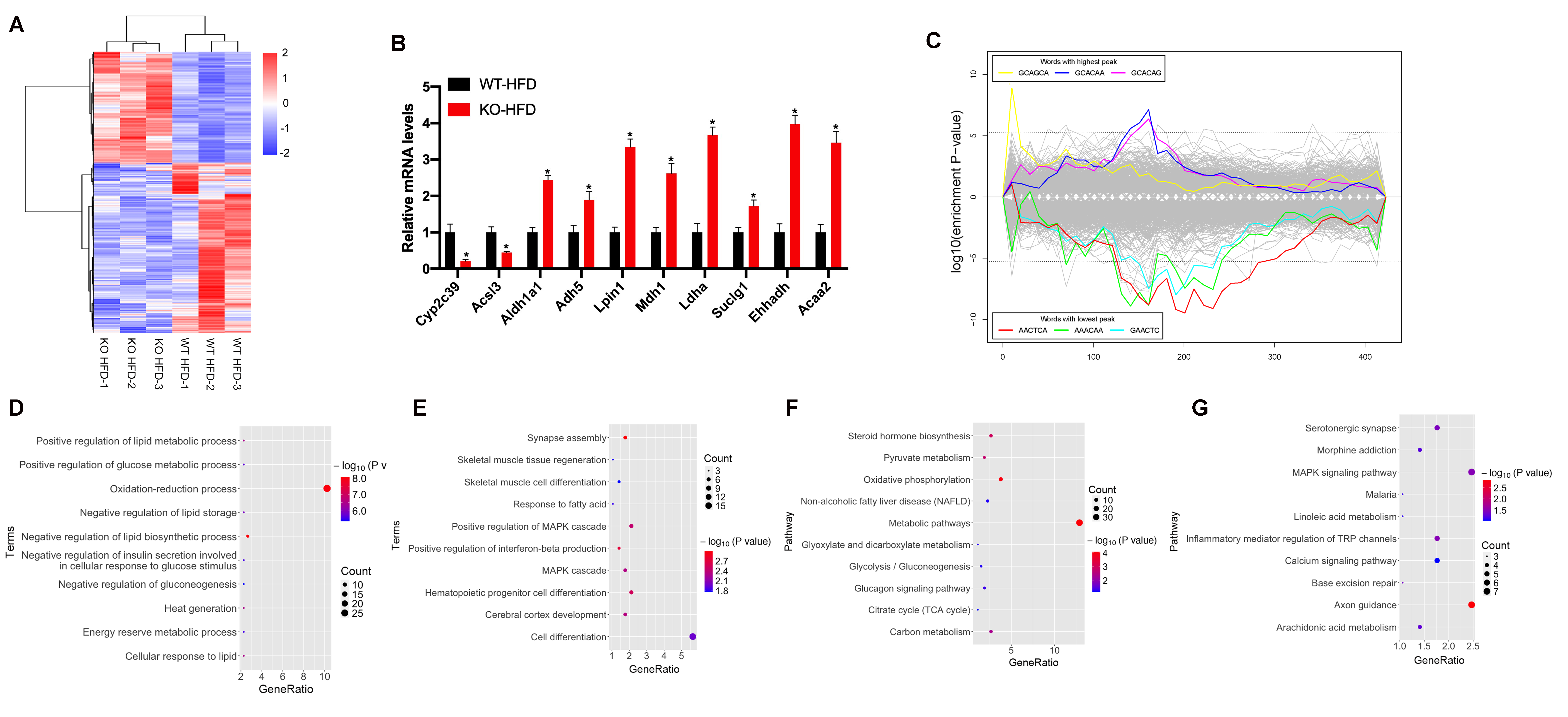

Supplement: Supplementary Figure 4 — RNA-seq revealed differential gene expression in KO-HFD mouse livers compared with that of in WT-HFD. (A) Heatmap and hierarchical clustering of 761 significantly altered genes in livers from WT-HFD and KO-HFD mice (n = 3). The blue-to-red colors of the heatmap are linearly mapped to the Z-scores, which range from −2 to 2. (B) The expression levels of 10 selected genes from RNA-seq in WT-HFD and KO-HFD mice livers by RT-qPCR analyses are shown. (C) A sylamer plot of motif enrichment in the 3′-UTRs of the dysregulated mRNAs assayed by RNA-seq. (D) Gene Ontology (GO) biological process analyses of significantly upregulated differential expressed genes (DEGs). (E) GO biological process analyses of significantly downregulated DEGs. (F) Kyoto Encyclopedia of Genes and Genomes (KEGG) pathway analyses of significantly upregulated DEGs. (G) KEGG pathway analyses of significantly downregulated DEGs. [file Image_4.TIF]
